# Undifferentiated Pleomorphic Sarcoma: A Diagnosis of Exclusion

**DOI:** 10.7759/cureus.85786

**Published:** 2025-06-11

**Authors:** Kyle W Eaton, Emily Jagenburg, Arian Pakray, Vikram Kinni, Benjamin Pomerantz

**Affiliations:** 1 Diagnostic Radiology, Henry Ford Health System, Southfield, USA; 2 Radiology, Oakland University William Beaumont School of Medicine, Rochester, USA; 3 Interventional Radiology, Henry Ford Health System, Southfield, USA

**Keywords:** arteriovenous malformation, hematoma, sarcoma, soft tissue hematoma, soft tissue tumors, undifferentiated pleomorphic sarcoma

## Abstract

A 43-year-old male patient presented with a progressively enlarging mass in his right lateral tibia over the course of one month. The mass measured 4 x 7 cm in size on examination. Aspiration of the lesion was performed, revealing 30 cc of dark blood, leading to an initial diagnosis of hematoma. Three months later, the mass had enlarged with mild pain. Physical examination revealed a 12 x 9 cm fluctuant mass on the right lateral lower leg, which was warmer than surrounding skin and tender upon palpation. Subsequent imaging was ambiguous, with a broad differential diagnosis consisting of expanding hematoma, abscess, neoplasm, and arteriovenous malformation (AVM). A biopsy was performed revealing pleomorphic cells consistent with an undifferentiated pleomorphic sarcoma (UPS). This case report highlights the challenges of diagnostic workup, management, and follow-up of an unusual presentation of a UPS.

## Introduction

Soft tissue masses in the extremities have a broad differential, ranging from benign hematomas to malignant sarcomas. Undifferentiated pleomorphic sarcoma (UPS) comprise 1% of all malignancies, with UPS accounting for 5-10% of soft tissue sarcomas [1,2]. These tumors occurs most frequently in the adult population, with the most common locations being the retroperitoneum and extremities [3]. Most cases present symptomatically due to progressive soft tissue swelling, pain, and, occasionally, pathologic fractures.

This report describes a case of a 43-year-old male patient with a one-month history of right lateral lower leg swelling. The diagnosis was made using a combination of ultrasound, CT, MRI, angiography, and eventual biopsy. UPS is primarily a diagnosis of exclusion, requiring histopathologic confirmation [1,2,4-7]. Management of UPS is dependent on tumor staging, with treatment often involving perioperative chemotherapy, en-bloc excision, and adjuvant radiation therapy to prevent local recurrence and mitigate metastatic disease [6,7]. This case highlights the importance of diagnostic imaging evaluation and tissue sampling in establishing a diagnosis.

## Case presentation

Clinical history

**Figure 1:**
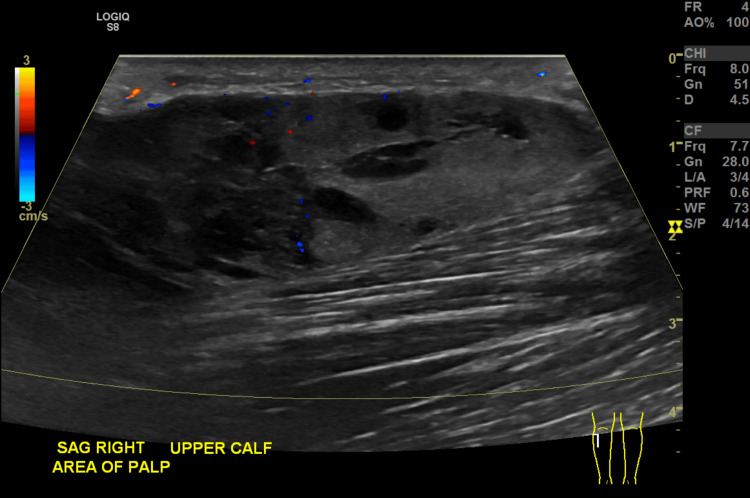
Right lower extremity ultrasound with color Initial imaging of a 9.1 × 2.2 × 4.4 cm heterogeneous, mostly hypoechoic mass without significant internal vascular flow overlying the imaged calf muscles, suggestive of hematoma.

**Figure 2:**
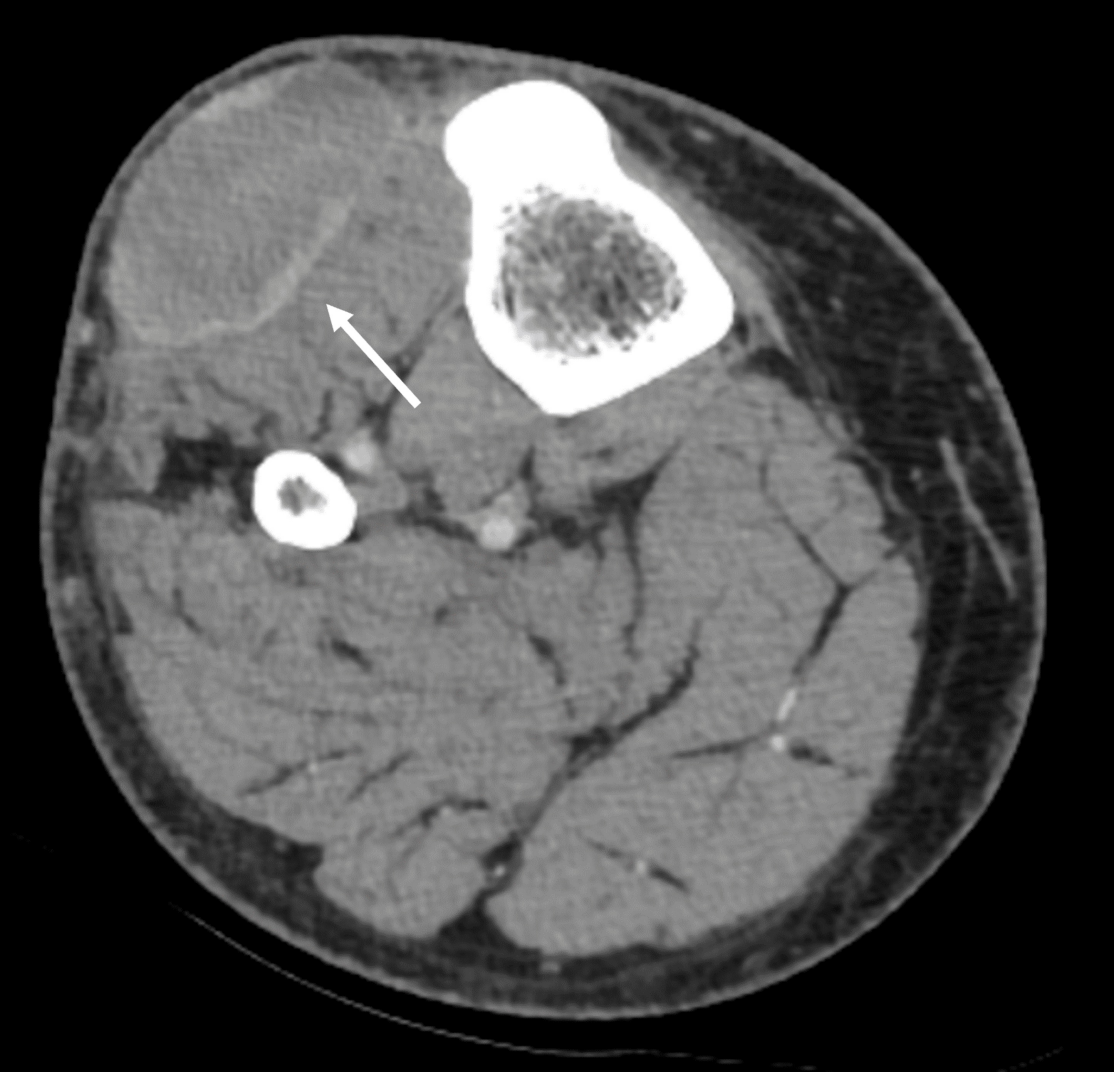
Contrast-enhanced CT of the right lower extremity 8.2 × 1.6 × 5.7 cm mass-like area (white arrow) with central fluid attenuation, septations, and peripheral enhancement located in the soft tissues lateral to the proximal tibia within the subcutaneous tissues overlying the musculature. There is sparing of the adjacent tibial cortex.

**Figure 3:**
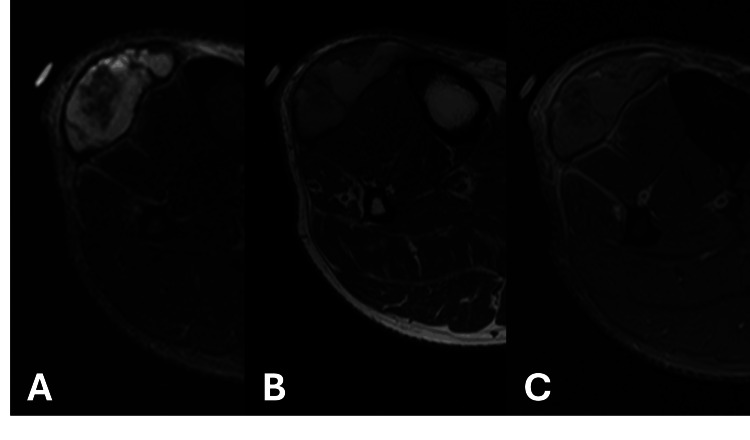
MRI of the right lower extremity with and without contrast A) STIR sequence shows a mixed signal intensity, multilobulated 4.5 × 3.1 × 8.6 cm mass lesion (transverse × anteroposterior × craniocaudal) within the deep subcutaneous fat overlying the extensor/anterior compartment muscles in the proximal leg. There is normal marrow signal of the adjacent tibia; B) There is T1 hyperintensity with thickened, hypointense outer rim; C) Post-contrast T1 demonstrates an enhancing outer wall without internal nodular or thick septal enhancement. STIR: Short T1 inversion recovery

A 43-year-old male patient with past medical history of hypertension and diabetes presents to the emergency department (ED) with a chief complaint of a progressively enlarging mass in the right lower leg. The mass was initially evaluated 10 weeks prior for the same issue. An ultrasound and CT were obtained at that time with finds consistent with a hematoma (Figures 1, 2). Additionally, an MRI was obtained as a neoplastic process could not be definitively excluded (Figure 3A-C). The patient was discharged with return precautions and a recommendation for follow up imaging to ensure resolution. Over the following few weeks, the mass continued to grow and became painful. He was urgently referred to the ED by his primary care physician.

The patient reports no history of trauma to the area. He does not take any blood thinners and has no known conditions affecting connective tissues, blood clotting, or bleeding tendencies. He describes tenderness over the mass but denies any associated redness, warmth, or drainage. Systemic symptoms such as fever or chills are absent. A full review of systems was completed with no other significant findings noted.

Physical exam and laboratory findings

The patient's blood pressure measures 143/67 mmHg, with a heart rate of 90 beats per minute and a respiratory rate of 17 breaths per minute. His temperature is 36.7°C, and oxygen saturation levels are at 95%. The patient weighs 120 kg, resulting in a BMI of 37 kg/m².

Upon general examination, the patient appears well-nourished and is in no acute distress. Examination of the extremities reveals a palpable mass measuring 13 x 4 x 10 cm located on the lateral aspect of the right lower leg. The mass is mildly tender to the touch, but there are no signs of erythema, induration, or drainage. Furthermore, there are no neurological deficits or vascular abnormalities noted.

Laboratory results indicate leukocytosis. There is no evidence of significant anemia. Further pertinent laboratory values are summarized in Table 1.

**Table 1:** Laboratory values WBC: White blood cell; CRP: C-reactive protein; BUN: Blood urea nitrogen

Test	Initial visit	Follow-up	Reference range
WBC count	11.93 K/mcL	12.73 K/mcL	4.2-5.9 K/mcL
Hemoglobin	15.8 g/dL	14.7 gm/dL	12.0-16.0 g/dL
Hematocrit	48.8%	44%	36-46%
Platelet Count	186 K/mcL	264 K/mcL	150-450 K/mcL
D-Dimer	<200 ng/mL	N/A	0-0.5 ng/mL
CRP	7.5 mg/L	N/A	0-10 mg/L
BUN	15 mg/dL	21 mg/dL	8-20 mg/dL
Creatinine	0.8 mg/dL	0.8 mg/dL	0.7-1.2 mg/dL

Additional imaging findings and management

**Figure 4:**
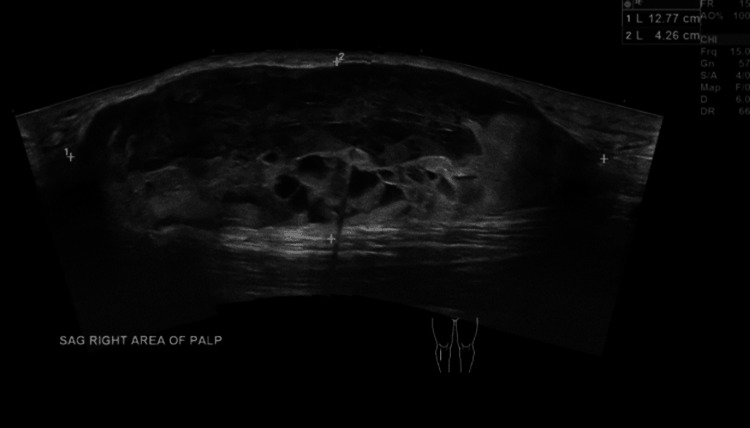
Ultrasound of the right lower extremity Two months after initial imaging. There is now a heterogeneous mass measuring 12.8 × 4.3 × 9.4 cm which appears external to the muscle fascia in the anterior right leg. The size has increased since the prior MRI.

**Figure 5:**
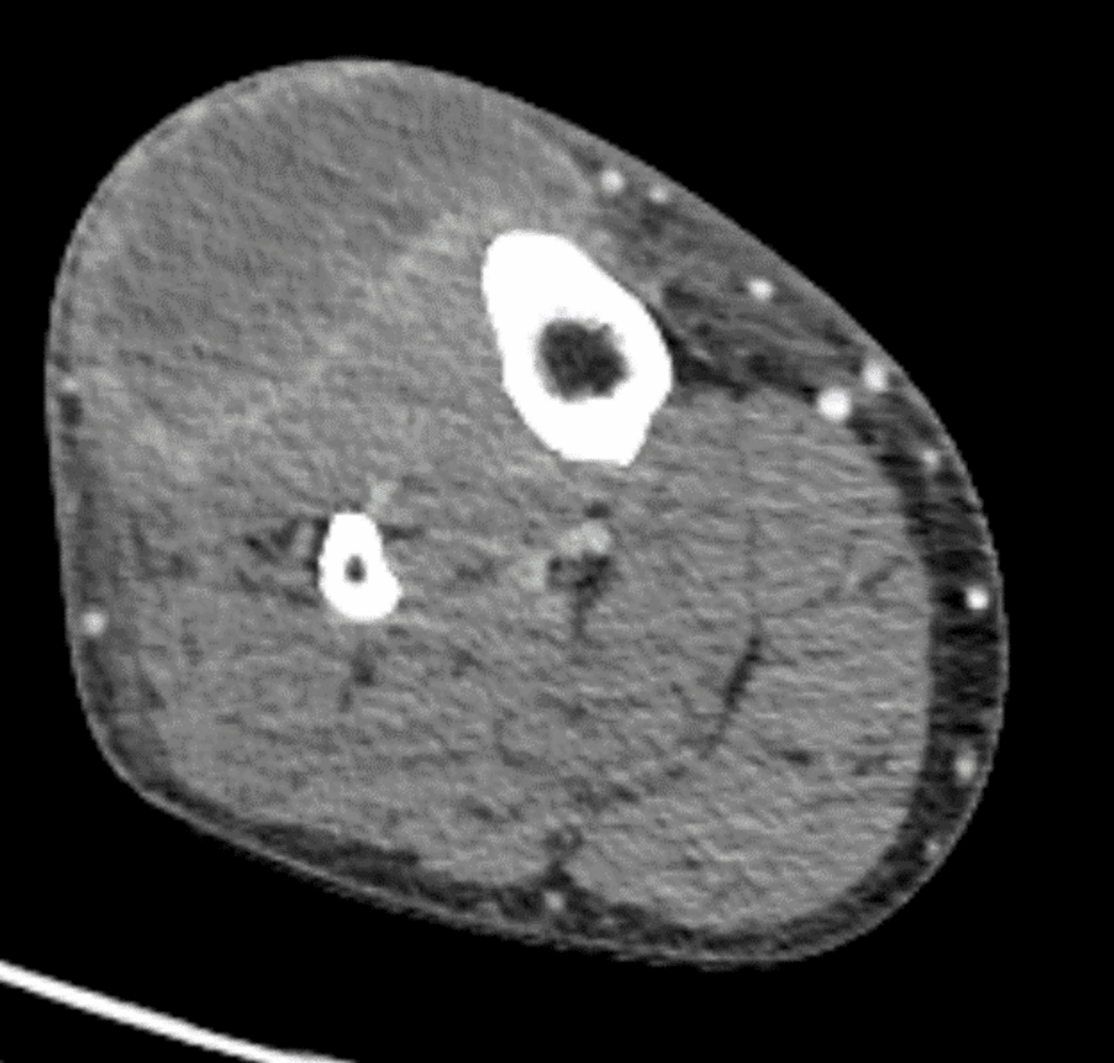
CT angiography of the right lower leg Heterogeneous-appearing mass within the anterior right lower leg has increased in size from November 21, 2023

A follow-up ultrasound of the mass was obtained demonstrating interval increase in size and new areas of internal vascularity (Figure 4). Based on the clinical and imaging findings, the working differential diagnosis included abscess, expanding hematoma, arteriovenous malformation (AVM), or neoplasm. A CT angiogram of the right lower extremity was ordered to evaluate the patient for an AVM (Figure 5). The ED consulted general surgery, and aspiration was attempted. Only a small amount of serosanguinous fluid was obtained, making an abscess or hematoma less likely. Vascular surgery and interventional radiology were consulted, and the decision was made to perform an angiogram of the right lower extremity with embolization of the suspected AVM. 

**Figure 6:**
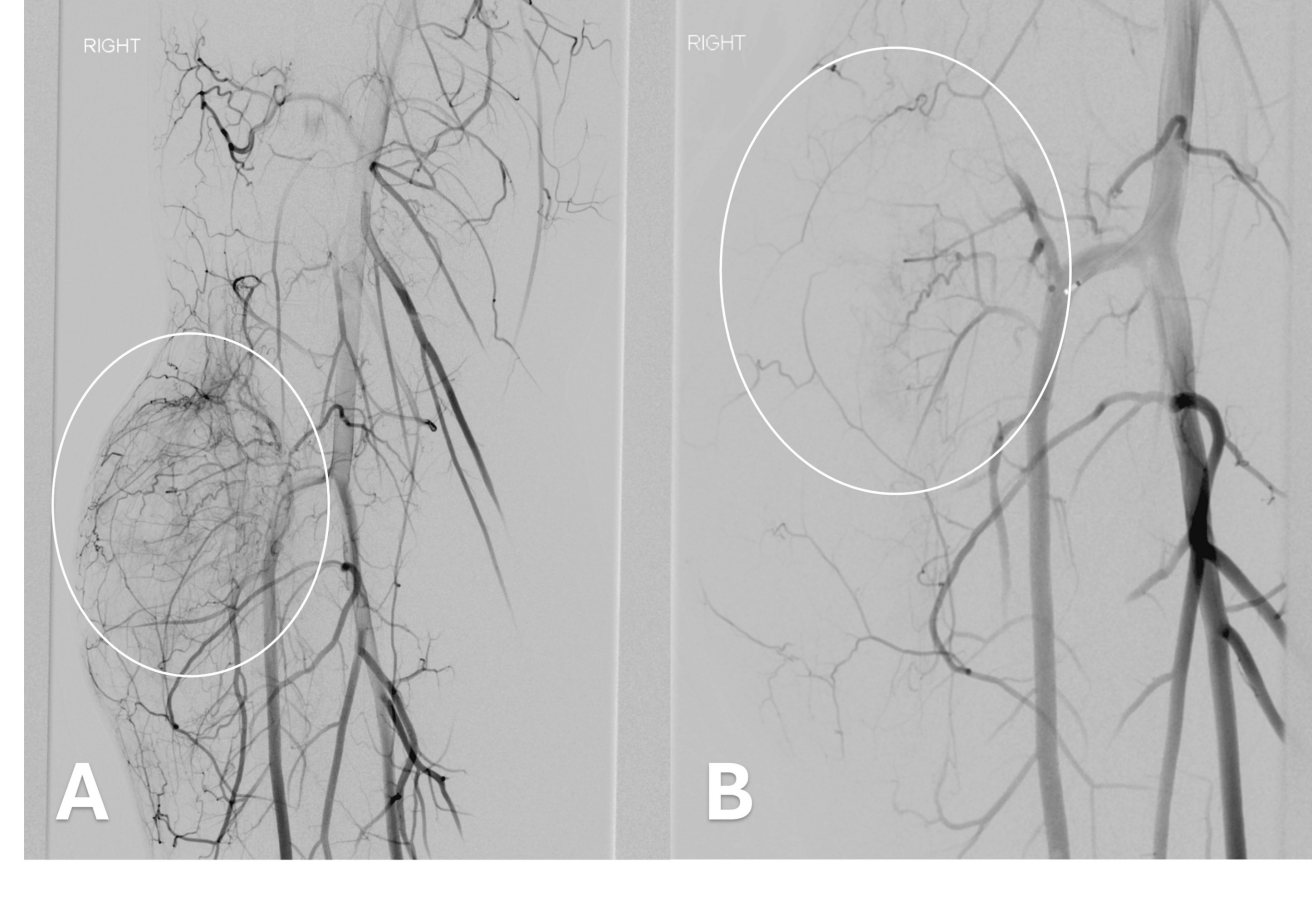
Right lower extremity angiogram A) Hypervascular mass in the lateral right lower leg supplied by arterial branches of the anterior tibial artery. No evidence of venous shunting seen; B) Post embolization with no arterial flow into the mass

The next day, selective angiography of the right femoral artery with digital subtraction confirmed a hypervascular mass in the lateral aspect of the lower leg correlating with the previously identified mass (Figure 6A, 6B). There was no evidence of venous shunting to indicate a suspected AVM. The findings were more suspicious for hypervascular neoplasm. After a multidisciplinary discussion with the patient, general and vascular surgery, the decision was made to embolize and biopsy the soft tissues mass which was now felt to represent a neoplasm and not an AVM.

An angiogram of the right anterior tibial artery revealed three branches supplying the tumor, which were embolized with 400-500 µm embolic particles following a core biopsy under ultrasound guidance. The biopsy tract was stained with India ink to aid in future surgical resection. The patient was discharged the next day with an orthopedic oncology follow-up at an outside institution.

Pathology findings and outcome

**Figure 7:**
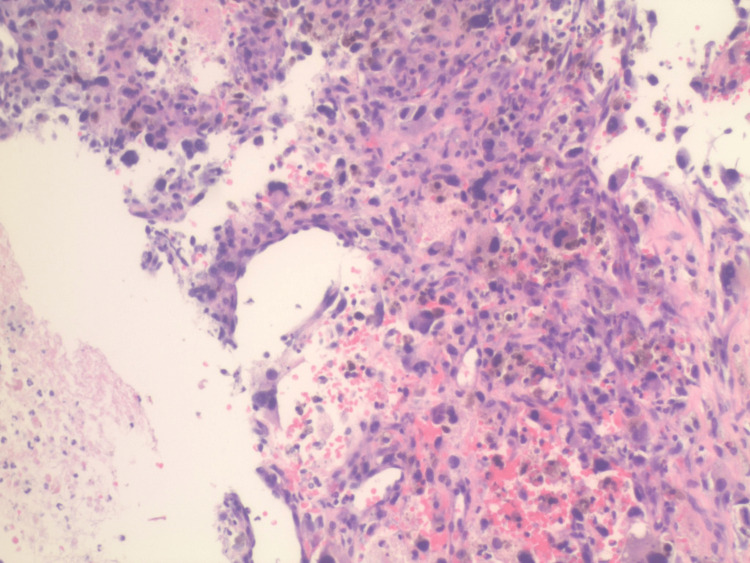
Core needle biopsy of the lower extremity mass. H&E staining X20 magnification. H&E: Hematoxylin and eosin

**Figure 8:**
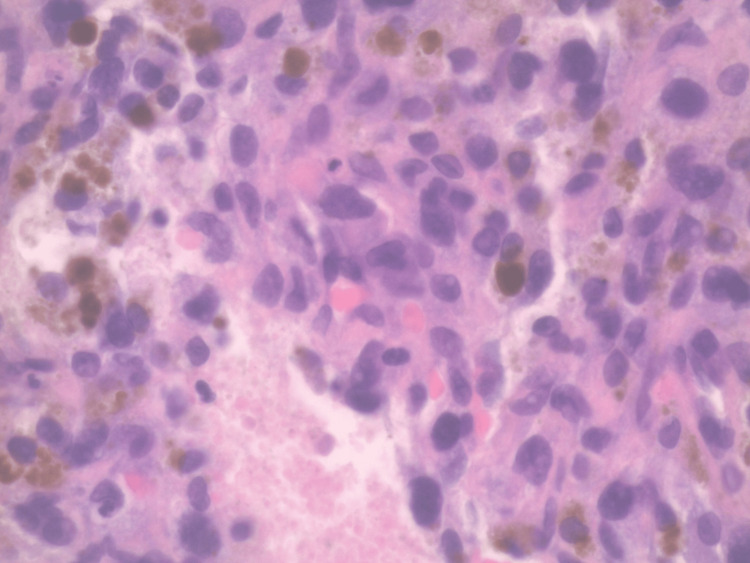
Core needle biopsy of the lower extremity mass. H&E staining X40 magnification. H&E: Hematoxylin and eosin

Needle core biopsy of the right lower extremity mass displayed features consistent with a high-grade UPS. The specimen consisted of neoplastic spindle and pleomorphic cells in the background of substantial blood and inflammatory cells. The tumor cells exhibited irregular and hyperchromatic nuclei, and atypical mitoses were also noted. A review of the immunohistochemical stains showed that the cells of interest are negative for CD31, smooth muscle actin, SATB2, PAX5, CD45, ALK1, CD34, desmin, HHV8, pancytokeratin, S100, SOX10, MUM1, CD138 (Figures 7, 8).

The patient was transferred to a nearby hospital for surgical resection and evaluation for distant metastasis. No evidence of distant metastasis was found. The patient underwent a free right latissimus dorsi flap for coverage of the right lower extremity following radical resection of the right calf sarcoma, achieving clear oncologic margins. Right inguinal lymph node dissection was performed and surgical incision was managed post-operatively with a negative pressure wound vacuum-assisted closure (VAC). The tumor was staged as grade 3 pT3 pN0 with all resection margins negative with less than 0.1 cm from the deepest margin.

On post-op day 5 (POD 5), the patient’s wound irrigation and debridement revealed a clean wound bed. The surgical incision was then closed both deep and superficially, and the wound VAC was exchanged. On POD 8, the patient underwent plastic reconstructive surgery with placement of a 21 x 20 cm split thickness skin graft over the muscle flap, followed by a reapplication of the wound VAC. The patient remained hemodynamically stable and was discharged home on POD 14.

## Discussion

Epidemiology

UPS, previously known as malignant fibrous histiocytoma, is a subcategory of soft tissue sarcomas distinguished by non-specific cytology differentiation and heterogeneous histologic appearance. It represents approximately 5-10% of soft tissue sarcomas in adults and typically occurs in individuals older than 50 [1,2]. Although rare among all tumor types, UPS is among the most common types of soft tissue sarcoma. There is no significant gender predilection [2]. The most common anatomical locations for UPS are the lower extremities, particularly the thigh, followed by the upper extremities and retroperitoneum [3,4]. While UPS can rarely arise from bones, the overwhelming majority originate from deep soft tissue structures [5]. Most cases are primary tumors, although approximately 30% are secondary to previous radiation therapy or pre-existing conditions [6,7]. Most cases of UPS are high-grade (3 and 4) upon diagnosis and exhibit aggressive behavior with early metastasis in disease course. Metastasis is present at the time of diagnosis in 30-50% of cases [8]. Local recurrence is not uncommon despite aggressive management (e.g., perioperative chemotherapy and en-bloc surgical resection). 

Clinical features

Clinical presentation of soft tissue UPS typically involves a rapidly growing, often painless mass, though it may occasionally present with discomfort or tenderness due to compression of surrounding structures [3,4]. The thigh and retroperitoneum are common locations, but these tumors can occur in virtually any soft tissue location [3]. The tumor mass frequently exhibits heterogenous internal composition, including areas of necrosis and/or hemorrhage [6]. The lesions tend to grow rapidly, leading to symptoms related to mass effect or local invasion rather than bone destruction. Unlike bone-originating sarcomas, soft tissue UPS does not typically present with cortical bone destruction or medullary involvement until there is advanced local invasion [6,7]. It is estimated that UPS has corresponding metastases in approximately 30-50% of cases. The most common site of metastasis is the lungs [6,7,9].

Risk factors

The most significant risk factor for high-grade UPS is age. Patients who are over 60 years old have an increased risk for adverse outcomes, mortality, and local recurrence [10]. Tumor size is associated with a worse prognosis, such that there is a higher likelihood for recurrence with tumors greater than 5 cm and an increased risk of mortality for tumors greater than 7 cm. Pleomorphic sarcomas that invade beyond subcutaneous fat and or deep within tissue also have a higher rate of recurrence and metastatic potential through lymphovascular invasion, leading to worse prognosis and mortality [4]. UPS is associated with other conditions and can arise secondary to bone infarction, Paget disease, previous bone irradiation, metallic hardware, and Hardcastle syndrome [6,7,9].

Diagnosis

The diagnosis of UPS typically relies on clinical assessment, imaging studies, and biopsy confirmation [2,4]. Histologically, UPS features include pleomorphic, spindle-to-epithelioid-shaped cells organized in a storiform or fascicular growth pattern [6,7]. Importantly, these tumors lack osteoid or cartilage matrix production, distinguishing them from many osteosarcomas and other primary bone malignancies. Immunohistochemistry does not provide definitive diagnostic markers, though vimentin positivity is commonly observed [7]. Radiologic imaging, particularly MRI, typically reveals a large, well-circumscribed, heterogeneous soft tissue mass without intrinsic bone involvement [6,8]. UPS lesions generally demonstrate intermediate intensity on T1-weighted images and high intensity on T2-weighted sequences, with variable enhancement patterns reflecting areas of necrosis, hemorrhage, or myxoid change [6]. Osteolytic features, cortical destruction, and calcifications are uncommon unless secondary local bone invasion has occurred, which usually indicates an advanced stage rather than primary tumor characteristics [6,9].

This particular case ultimately required the use of invasive angiography to rule out the leading working diagnosis of AMV. This lead to a rather unique scenario as biopsy of a suspected AMV is generally discouraged due to the high risk of bleeding. After an informed discussion with the patient and other members of the care team, the decision was made to perform a tissue biopsy to establish the diagnosis.

Management

The corresponding TNM staging typically dictates the management. There is a 38.3% 5-year and a 30.5% 10-year survival rate [1,7,11]. There is an increased survival rate in cases without metastasis. Secondary UPS typically has a worse prognosis compared to the primary counterpart [7,8]. Treatment options include surgery with radical resection with perioperative chemotherapy. Neoplasms that are unresectable or have metastatic disease upon presentation can be managed with radiotherapy for palliative measures [4,6-10]. Cases originating in the extremities are usually amenable to limb-sparing treatment [6,7].

## Conclusions

This case report underscores the challenges in diagnosing soft tissue sarcomas. The initial patient presentation of a relatively small mass in the right lower lateral tibia, understood to be hematoma based on imaging and clinical presentation, progressed in size and required further imaging studies, biopsy, and pathology evaluation for the official diagnosis of high-grade UPS. This case was further confounded by the lack of osseous involvement, which, if present, may have led to earlier identification and biopsy. A multidisciplinary approach integrating diagnostic imaging, interventional radiology, and pathological analysis proved to be essential in facilitating a timely intervention in managing this aggressive atypical soft tissue mass. 

Careful attention to detail, early recognition, and a comprehensive clinical evaluation are imperative in characterizing rare soft tissue sarcomas. This report reinforces and describes the importance of precision and maintaining a high index of suspicion in diagnosing tumors that may appear ambiguous but directly dictate the direction of treatment and management and long-term patient outcomes. Due to the aggressive nature of these sarcomas, ensuring adequate follow-up and care is integral to improving the prognosis of patients with rare diagnoses.
